# Effect of Tissue-Selective Estrogen Complex on Hip Structural Geometry in Postmenopausal Women: A 12-Month Study

**DOI:** 10.3389/fendo.2021.649952

**Published:** 2021-03-11

**Authors:** Bo Mi Kim, Sung Eun Kim, Dong-Yun Lee, DooSeok Choi

**Affiliations:** Department of Obstetrics and Gynecology, Samsung Medical Center, Sungkyunkwan University School of Medicine, Seoul, South Korea

**Keywords:** tissue-selective estrogen complex (TSEC), bone mineral density, hip structural analysis, menopause, hormone therapy (HT)

## Abstract

**Background:**

Hip structural analysis (HSA) is a method for evaluating bone geometry reflecting bone structural and biomechanical properties. However, tissue-selective estrogen complex (TSEC) treatment effects on HSA have not been investigated.

**Objective:**

This study was performed to evaluate the effect of TSEC treatment on hip geometry in postmenopausal Korean women. The treatment was given for 12 months, and hip geometry was measured by HSA.

**Materials and Methods:**

A total of 40 postmenopausal women who received TSEC containing conjugated estrogen 0.45 mg and bazedoxifene 20 mg for treating vasomotor symptoms were included in this retrospective cohort study. The changes in bone mineral density and parameters of HSA including the outer diameter, cross-sectional area, cross-sectional moment of inertia, cortical thickness, section modulus, and buckling ratio as determined by dual-energy X-ray absorptiometry were compared before and after 12 months of TSEC treatment.

**Results:**

Mean age and years since menopause were 55.1 and 4.5 years, respectively. Total hip bone mineral density significantly increased by 0.74% after treatment (P=0.011). The changes in HSA were mainly demonstrated in the narrow femoral neck: cross-sectional area (P=0.003) and cortical thickness (P<0.001) increased significantly. For the shaft region, only SM decreased significantly after treatment (P=0.009). However, most parameters did not change significantly with TSEC treatment in the intertrochanteric and shaft regions.

**Conclusions:**

Our findings demonstrate that 12 months of TSEC treatment could improve bone geometry as measured by HSA. The findings suggest that TSEC might be an interesting option for the prevention of fracture as well as osteoporosis in postmenopausal women.

## Introduction

Menopausal hormone therapy (MHT), estrogen alone, or estrogen-progestin combination, is effective for the prevention of osteoporosis and fractures in postmenopausal women (1, 2). However, occurrence of adverse side effects and concerns about risks associated with MHT have deterred use of MHT for this purpose. Experimental evidence suggests that most of the side effects and risks are related to the progestogen component, and a combined therapy of conjugated estrogen (CE) and bazedoxifene was developed recently and used worldwide as an alternative to conventional MHT. This new progestin-free tissue-selective estrogen complex (TSEC) would be a useful MHT option especially in women who cannot tolerate the side effects related to progestogens or who have a higher risk for breast cancer (1), and also showed beneficial effects on bone mineral density (BMD) in previous studies (3–5).

Estrogen regulates bone metabolism *via* effects on osteocytes, osteoblast, and osteoclasts, and its main effect is inhibition of bone remodeling (6). MHT reduced all major osteoporotic fractures including hip and non-vertebral fractures (7, 8), and bazedoxifene reduced spinal fractures and even prevented hip fractures in postmenopausal women at higher risk of hip fracture (9). Therefore, TSEC, a combination of these two regimens, can also be expected to have beneficial effects on fracture risk. However, fracture risk has not been adequately assessed for low doses of CE (< 0.625 mg), although the effect of estrogen on bone is dose-dependent. Moreover, the effect of TSEC on fracture risk has never been evaluated because the study duration was short (12–24 months) and the subjects were young (subject mean age was in their 50s) in previous studies (3–5).

Hip structural analysis (HSA) is a method for evaluating bone geometry reflecting bone structural and biomechanical properties using dual energy X-ray absorptiometry (DXA) scan of the proximal femur. HSA is a software method to extract information, a line of pixel values across the proximal femur bone axis, from image data of DXA bone mass (10). The profile of a mass projection could be used to estimate geometric relevance. In spite of several limitations such as high correlation with BMD or 2D nature (11, 12), HSA has been applied to assess bone mechanical strength, and therefore, to evaluate the effect of osteoporosis treatment on fracture risk (13–15). However, TSEC treatment effects on HSA have not been investigated.

Therefore, this study evaluated the change in HSA after 12 months of TSEC treatment in postmenopausal Korean women.

## Materials and Methods

### Study Population

All postmenopausal women who received MHT for relieving vasomotor symptoms at the Menopause Clinic at Samsung Medical Center were considered for inclusion. Menopause was diagnosed when women had no spontaneous menstruation over 12 months without any specific cause for amenorrhea.

The inclusion criteria were: postmenopausal women (1) with an intact uterus, (2) who received TSEC, (3) who were over 40 years old, (4) who were diagnosed with osteopenia at the femoral neck or total hip using DXA, and (5) who had results of bone densitometry and geometry before and after 12 months of TSEC use.

Exclusion criteria were: (1) use of MHT other than TSEC; (2) use of any medication that could affect bone metabolism, e.g., bisphosphonate, glucocorticoids, anti-convulsants, or heparin; (3) having a history of any disease that could affect bone metabolism, e.g., hyperthyroidism or hyperparathyroidism; (4) having been lost to follow-up before 12 months of TSEC treatment; and (5) having a history of hip fracture.

A total of 40 postmenopausal women were included for the analysis in this retrospective cohort study. The study protocol was approved by the Institutional Review Board of Samsung Medical Center, Seoul, Korea. Informed consent from participants was exempted by the Institutional Review Board because of the retrospective nature of the analysis.

### Treatment and Measurement

Women received TSEC (Duavive^®^, Pfizer Ireland Pharmaceuticals, Newbridge, Ireland) for relief of vasomotor symptoms based on the preferences of both patients and doctors. Each TSEC tablet contained CE 0.45 mg and bazedoxifene 20 mg.

The baseline characteristics of the study population, i.e., age, body mass index, years since menopause, and parity, were obtained from medical records. In addition, the results of bone densitometry and hip geometry were also obtained.

BMD was measured at the hip using DXA (Delphi Q; Hologic Inc., Bedford, MA, USA). The *in vivo* coefficient of variation was 1.0% for the hip. For the evaluation of hip bone geometry, the HSA™ program was used for three regions, the femoral narrow neck, intertrochanter, and shaft. These were displayed on the DXA image. At each region, the HSA™ program produced geometric parameters including the outer diameter (OD), cross-sectional area (CSA), cross-sectional moment of inertia (CSMI), cortical thickness (CT), section modulus (SM), and buckling ratio (BR).

### Statistical Analysis

All statistical analyses were performed using SPSS Statistics 25 software (SPSS Inc., Chicago, IL). Data are presented as mean ± standard error of the mean or number (percent). The normality assumption of the data was confirmed using the Levene’s test for homogeneity of variances before statistical analysis. Changes in BMD and various parameters of HSA were compared before and after treatment using paired t tests. In addition, differences in HSA parameters between responders (no change or increase in BMD from baseline at 12 months) and non-responders (decrease in BMD from baseline at 12 months) were compared by t test or Mann–Whitney test. P < 0.05 was considered statistically significant.

## Results

**Table 1** demonstrates the baseline characteristics of the study participants. The mean age and years since menopause were 55.1 and 4.5 years, respectively. The mean body index was 21.5 kg/m^2^, and only two women were obese.

**Table 1:** Baseline characteristics of the study population.

Variables	N = 40
Age (year)	55.1 ± 0.5
Body mass index (kg/m^2^)	21.5 ± 0.4
Age at menopause (year)	50.7 ± 0.5
Years since menopause (year)	4.5 ± 0.4
Age at menarche (year)	14.5 ± 0.3
Parity	1.9 ± 0.2
Nulliparous	4 (10)
Multiparous	36 (90)
Type of menopause	
Natural	40 (100)
Surgical	0

Data are presented as mean ± standard error or number (percent).

**Figure 1:**
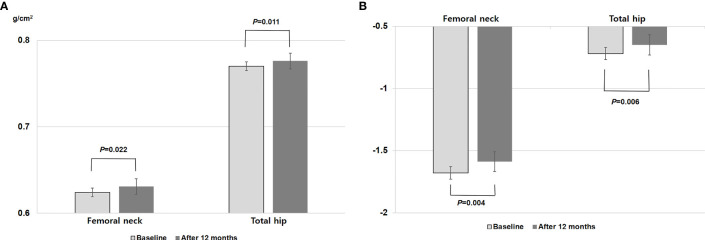
Changes in **(A)** bone mineral density and **(B)** T-score at the femoral neck and total hip after 12 months of TSEC treatment. P value by paired t test.

**Figure 1** depicts the change in BMD after 12 months of TSEC treatment. Both BMDs at the femoral neck (from 0.624 to 0.631 g/cm^2^, P = 0.022) and total hip (from 0.770 to 0.776 g/cm^2^, P = 0.011) increased significantly by 1.26% and 0.74%, respectively. In addition, T-score also improved significantly at the femoral neck (from -1.68 to -1.59, P = 0.004) and total hip (from -0.72 to -0.65, P = 0.006).

**Table 2** shows the changes in various parameters of HSA after 12 months of TSEC treatment. For the narrow neck of the femur, CSA (P = 0.003) and CT (P<0.001) increased significantly. In addition, although not statistically significant, CSMI and SM tended to increase and BR tended to decrease. However, OD did not change. For the intertrochanteric region, women had a wider diameter after treatment (P = 0.041). However, other parameters such as CSA, CSMI, SM, CT, and BR did not change significantly after treatment. For the shaft region, only SM decreased significantly after treatment (P = 0.009), and OD, CSA, CSMI, CT, and BR did not differ.

**Table 2:** Changes in parameters of hip structural analysis at three regions after 1 year of TSEC treatment.

Site	Variables	Baseline	After treatment	*P*-value
Narrow neck	OD (cm)	3.28 ± 0.045	3.25 ± 0.036	0.380
	CSA (cm^2^)	2.41 ± 0.026	2.46 ± 0.025	**0.003**
	CSMI (cm^4^)	2.06 ± 0.042	2.09 ± 0.042	0.075
	SM (cm^3^)	1.14 ± 0.019	1.16 ± 0.017	0.055
	CT (cm)	0.15 ± 0.002	0.16 ± 0.002	**<0.001**
	BR	12.4 ± 0.324	11.9 ± 0.244	0.074
Intertrochanter	OD (cm)	5.40 ± 0.041	5.51 ± 0.054	**0.041**
	CSA (cm^2^)	4.18 ± 0.060	4.27 ± 0.061	0.105
	CSMI (cm^4^)	11.06 ± 0.273	11.52 ± 0.285	0.061
	SM (cm^3^)	3.48 ± 0.076	3.57 ± 0.070	0.088
	CT (cm)	0.35 ± 0.006	0.36 ± 0.007	0.318
	BR	9.20 ± 0.176	9.21 ± 0.200	0.918
Femur shaft	OD (cm)	2.86 ± 0.026	2.88 ± 0.026	0.274
	CSA (cm^2^)	3.64 ± 0.045	3.63 ± 0.051	0.507
	CSMI (cm^4^)	2.94 ± 0.069	2.90 ± 0.061	0.166
	SM (cm^3^)	1.97 ± 0.032	1.93 ± 0.030	**0.009**
	CT (cm)	0.49 ± 0.010	0.49 ± 0.011	0.187
	BR	3.10 ± 0.095	3.19 ± 0.120	0.104

Data are presented as mean ± standard error. P-value by paired t test. NS, not significant; TSEC, tissue-selective estrogen complex; OD, outer diameter; CSA, cross-sectional area; CSMI, cross-sectional moment of inertia; SM, section modulus; CT, cortical thickness; BR, buckling ratio.

In bold: statistically significant p-values.

**Table 3** presents the changes in HSA according to BMD response. Responders were women with no change or increase in total hip BMD after treatment. Because the number of each group was relatively small, some parameters in **Table 3** did not meet the normality assumption. In these contexts, t test or Mann–Whitney test were used where indicated. For the narrow femoral neck, percent changes in OD, CSA, and CSMI were significantly higher in responders than in non-responders. Values were significantly decreased in OD and CSMI or only slightly increased in CSA in non-responders after treatment. Although not statistically significant, BR also decreased in responders in contrast to a minimal increase in non-responders. For the intertrochanteric region, OD and BR decreased in responders and increased in non-responders, and the changes were significantly different between responders and non-responders (P = 0.031 for OD and P=0.026 for BR). For the shaft region, CSA and CT increased in responders and decreased in non-responders after treatment, showing a significant difference of percent changes between the two groups (P = 0.005 for CSA and P = 0.042 for CT).

**Table 3:** Percent changes in parameters of hip structural analysis at three regions after 1 year of TSEC treatment, according to the response of bone mineral density at the total hip.

Site	Variables	Responder (n = 26)	Non-responder (n = 14)	*P*-value
Narrow neck	OD (cm)	3.06 ± 0.75	−4.33 ± 1.37	**0.001**
	CSA (cm^2^)	3.83 ± 0.77	0.38 ± 0.85	**0.005**
	CSMI (cm^4^)	4.47 ± 1.43	−0.13 ± 1.47	**0.031**
	SM (cm^3^)	2.98 ± 1.17	2.40 ± 2.36	0.827
	CT (cm)	1.53 ± 1.65	5.81 ± 2.34	0.142
	BR	−6.18 ± 3.01	0.40 ± 2.32	0.091
Intertrochanter	OD (cm)	−0.01 ± 0.99	4.01 ± 1.49	**0.031**
	CSA (cm^2^)	2.11 ± 1.30	2.72 ± 2.34	0.820
	CSMI (cm^4^)	2.03 ± 2.57	8.26 ± 3.94	0.194
	SM (cm^3^)	2.17 ± 1.77	4.16 ± 2.49	0.519
	CT (cm)	2.18 ± 1.85	1.62 ± 2.67	0.864
	BR	−2.14 ± 1.03	2.34 ± 1.62	0.026
Femur shaft	OD (cm)	0.14 ± 0.61	1.04 ± 0.73	0.349
	CSA (cm^2^)	0.95 ± 0.65	−1.68 ± 0.58	**0.005**
	CSMI (cm^4^)	−2.65 ± 1.46	0.35 ± 1.47	0.157
	SM (cm^3^)	−2.64 ± 0.99	−1.20 ± 1.00	0.315
	CT (cm)	0.70 ± 1.39	−3.42 ± 1.37	**0.042**
	BR	0.25 ± 2.02	5.93 ± 2.59	0.092

Data are presented as mean ± standard error. P-value by t test or Mann–Whitney test as indicated. NS, not significant. TSEC, tissue-selective estrogen complex.

Responder, no change or increase in bone mineral density from baseline at 12 months; non-responder, decrease in bone mineral density from baseline at 12 months.

OD, outer diameter; CSA, cross-sectional area; CSMI, cross-sectional moment of inertia; SM, section modulus; CT, cortical thickness; BR, buckling ratio.

In bold: statistically significant p-values.

## Discussion

This study evaluated the changes in BMD and HSA after 12 months of TSEC treatment in postmenopausal Korean women. This study demonstrated that, with TSEC treatment, hip BMD significantly increased, and some parameters of HSA were improved with TSEC treatment.

In the present study, total hip BMD significantly increased by 0.7% after treatment. This finding is similar to previous studies that demonstrated an approximate 1% (SMART-1), 0.84% (SMART-4), and 0.5% (SMART-5) increase of total hip BMD after 12 months of TSEC treatment (3–5). Since our study population was osteopenic postmenopausal women who were at low risk for bone loss or fracture, this amount of BMD increase could maintain women’s bone health (16). In addition, the percentage of total hip BMD responders was 65% in the present study. This is comparable to the 61.5% of SMART-4 (4) and 70% of SMART-1 responders (3). The amount of total hip BMD increase in the current study was similar to that seen with bazedoxifene alone (9). This suggests that the combination of bazedoxifene 20 mg and CE 0.45 mg did not produce an additional BMD gain by adding the estrogen component. One possible explanation is that, since both CE and bazedoxifene bind to the estrogen receptor and CE has a more potent effect, addition of bazedoxifene to CE may attenuate the BMD response (3, 5).

The risk of hip fracture is low in young postmenopausal women who usually take TSEC, and the study duration in previous studies regarding TSEC was too short (≤ 24 months). Therefore, fracture risk related to TSEC use has never really been addressed. In addition, either low-dose CE (0.45 mg) or bazedoxifene therapy has not been proven to reduce the hip fracture risk in postmenopausal women at low risk. In these contexts, HSA was assessed as a possible surrogate marker to evaluate beneficial effects of TSEC on fracture risk in this study.

In the present study, the changes in HSA parameters, indicating geometry-related improvement, were mainly observed in the narrow femoral neck region. The femoral neck is the most common location of fractures. Our findings are consistent with the 3-year retrospective study in postmenopausal women with osteoporosis (mean age: 67.4 years). Those researchers reported that treatment with bazedoxifene was associated with significant increases in CSA and SM in the narrow femoral neck and in CSA in the trochanter region. Also, almost no significant change in the shaft region compared with placebo was demonstrated (17). Besides bazedoxifene results, results from the following three studies regarding MHT are also similar to our study findings. In a prospective study analyzing about 600 current hormone users, CSA and CT increased compared to never users (18). In addition, in the 3-year randomized clinical trial in women over the age of 65 years, MHT using CE 0.625 mg (n = 93) increased CSA and SM in the narrow femoral neck region, and BR decreased after treatment (19). From the Women’s Health Initiative study (20), CSA and CT increased and BR decreased in the narrow neck region at year 1 among hormone users compared with placebo. Favorable changes in HSA parameters in the intertrochanter or shaft region were also not observed. No significant differences in the geometric changes were found between estrogen alone and estrogen-progestin treatment, suggesting that the effect of estrogen was responsible for the changes.

The beneficial effect of estrogen seems to be associated with increases in cortical thickness and section moduli *via* direct effects on bone (18). The load sensitivity can be increased by estrogen on bone tissue. In addition, estrogen has a positive effect on muscle strength; this may play an indirect role on bone strength (21). However, we could not conclude that the combination of bazedoxifene and CE had additional benefits on hip structural geometry compared with estrogen or bazedoxifene alone. Differences in treatment (dose, type, duration) and study population (age, baseline BMD, history of fracture, or other risk factors) have a considerable impact on bone health; therefore, the degree of beneficial effects will differ across studies.

This is the first study to evaluate the change in HSA with TSEC treatment in young osteopenic postmenopausal women. Although previous studies showed a significant increase in BMD and significant decreases in bone turnover markers, HSA has never been assessed in TSEC users. In addition, previous studies evaluated the effects of MHT on HSA in older women (over 65 years old); but, from recent guidelines, MHT is no longer recommended for bone health in older women (1, 2). The current main indication for MHT is young postmenopausal women having vasomotor symptoms who are younger than 60 years or within 10 years from menopause onset. This is similar to our study population. Therefore, the current study could provide real world evidence regarding TSEC on hip geometry.

However, this study had several limitations. This was not a prospective clinical trial, and duration of treatment and sample size may not have been adequate to assess the effects of TSEC on HSA. In a post-hoc power analysis, this study had a power of about 30% to detect differences in the changes of HSA parameters with an alpha error of 0.05. This study did not contain a control group who had osteopenia but did not use TSEC. However, it already has been shown that parameters of HSA did not change significantly in placebo group during one year (20). In addition, although intake of calcium and vitamin D was encouraged for all patients and daily supplementation was provided if necessary, we could not ascertain that all patients consumed sufficient calcium and vitamin D for maintaining bone health. Finally, the clinical implication of our findings is not clear in young postmenopausal women at low risk of hip fracture, and therefore, the results should be interpreted with caution.

In conclusion, this pilot study demonstrates that a combination of CE and bazedoxifene could enhance improvements in bone strength evaluated by HSA and suggests that TSEC may be a treatment option for prevention of fracture and osteoporosis in postmenopausal women. Further long-term, large-scale study is necessary to draw a clear conclusion.

## Data Availability Statement

The raw data supporting the conclusions of this article will be made available by the authors, without undue reservation.

## Ethics Statement

The studies involving human participants were reviewed and approved by Samsung medical center. Written informed consent for participation was not required for this study in accordance with the national legislation and the institutional requirements.

## Author Contributions

BK, SK, D-YL, and DC were responsible for the concept and design of the study, searching for and analyzing data, and the writing of the manuscript. All authors contributed to the article and approved the submitted version.

## Conflict of Interest

The authors declare that the research was conducted in the absence of any commercial or financial relationships that could be construed as a potential conflict of interest.
